# Serum dihydrotestosterone levels are associated with adverse myocardial remodeling in patients with severe aortic valve stenosis before and after aortic valve replacement

**DOI:** 10.1152/ajpheart.00288.2022

**Published:** 2022-10-07

**Authors:** Marie Schafstedde, Johannes Nordmeyer, Felix Berger, Christoph Knosalla, Philipp Mertins, Matthias Ziehm, Marie-Luise Kirchner, Vera Regitz-Zagrosek, Titus Kuehne, Milena Kraus, Sarah Nordmeyer

**Affiliations:** ^1^Department of Congenital Heart Disease and Paediatric Cardiology, German Heart Center Berlin, Berlin, Germany; ^2^Institute of Computer-assisted Cardiovascular Medicine, Charité-Universitätsmedizin, Berlin, Germany; ^3^German Center for Cardiovascular Research (DZHK), Berlin, Germany; ^4^Berlin Institute of Health at Charité-Universitätsmedizin Berlin, Berlin, Germany; ^5^Department of Cardiothoracic and Vascular Surgery, Deutsches Herzzentrum, Berlin, Germany; ^6^Proteomics Platform, Max Delbrück Center for Molecular Medicine in the Helmholtz Association, Berlin, Germany; ^7^Institute for Gender in Medicine, Center for Cardiovascular Research, Berlin, Germany; ^8^Digital Health Center, Hasso Plattner Institute for Digital Engineering, University of Potsdam, Potsdam, Germany

**Keywords:** aortic valve stenosis, cardiac remodeling, dihydrotestosterone, proteomics

## Abstract

Animal studies show a pivotal role of dihydrotestosterone (DHT) in pressure overload-induced myocardial hypertrophy and dysfunction. The aim of our study was to evaluate the role of DHT levels and myocardial hypertrophy and myocardial protein expression in patients with severe aortic valve stenosis (AS). Forty-three patients [median age 68 (41–80) yr] with severe AS and indication for surgical aortic valve replacement (SAVR) were prospectively enrolled. Cardiac magnetic resonance imaging including analysis of left ventricular muscle mass (LVM), fibrosis and function, and laboratory tests including serum DHT levels were performed before and after SAVR. During SAVR, left ventricular (LV) biopsies were performed for proteomic profiling. Serum DHT levels correlated positively with indexed LVM (LVMi, *R* = 0.64, *P* = 0.0001) and fibrosis (*R* = 0.49, *P* = 0.0065) and inversely with LV function (*R* = −0.42, *P* = 0.005) in patients with severe AS. DHT levels were associated with higher abundance of the hypertrophy (moesin, *R* = 0.52, *P* = 0.0083)- and fibrosis (vimentin, *R* = 0.41, *P* = 0.039)-associated proteins from LV myocardial biopsies. Higher serum DHT levels preoperatively were associated with reduced LV function (ejection fraction, *R* = −0.34, *P* = 0.035; circulatory efficiency, *R* = −0.46, *P* = 0.012; and global longitudinal strain, *R* = 0.49, *P* = 0.01) and increased fibrosis (*R* = 0.55, *P* = 0.0022) after SAVR. Serum DHT levels were associated with adverse myocardial remodeling and higher abundance in hypertrophy- and fibrosis-associated proteins in patients with severe AS. DHT may be a target to prevent or attenuate adverse myocardial remodeling in patients with pressure overload due to AS.

**NEW & NOTEWORTHY** Serum dihydrotestosterone (DHT) levels correlated positively with the degree of hypertrophy, fibrosis, and dysfunction from cardiac magnetic resonance imaging in female and male patients with aortic valve stenosis. Left ventricular proteome profiling had been performed in this patient cohort and an association between serum DHT levels and the abundance of the hypertrophy-associated protein moesin and the fibrosis-associated protein vimentin was found.

## Graphical abstract

**Figure:**
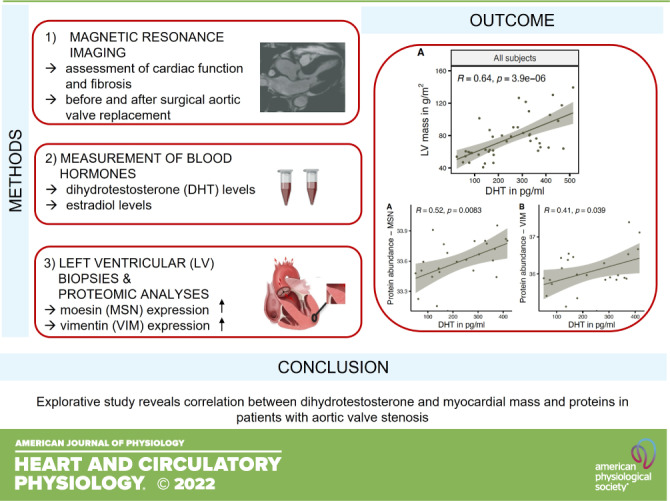

## INTRODUCTION

Several animal studies have described a relationship between testosterone, its active metabolite dihydrotestosterone (DHT), and cardiac hypertrophy (1, 2). Testosterone and DHT act via androgen receptors and have shown to induce an androgen receptor-dependent hypertrophic response in neonatal rat myocytes (3). The reduction of DHT levels via antiandrogen therapy with finasteride effectively attenuated cardiac hypertrophy and left ventricular (LV) dysfunction in female and male mice with pressure overload, improved cardiac function, and attenuated remodeling after myocardial infarction in mice (1, 2).

Androgen receptors are present in healthy male and female hearts, and higher free testosterone levels have been associated with elevated left ventricular muscle mass (LVM) (3, 4). Antiandrogenic therapy in male patients with prostate cancer and heart failure was associated with lower LV septal thickness, and in male patients with type I diabetes, testosterone serum levels were associated with higher LVM (5, 6).

In patients with severe aortic valve stenosis (AS), left ventricular hypertrophy (LVH), fibrosis, and dysfunction are common and associated with adverse outcomes (7–10). Several studies have shown that female patients with AS show less hypertrophy and better function, as well as less morbidity and mortality than male patients with AS (11, 12). In addition, it is known that postmenopausal women show lower indexed LVM (LVMi) and lower levels of testosterone and free testosterone (4). However, an association between DHT serum levels and the degree of LVH has not yet been investigated in female and male patients with severe AS.

The aim of the present study was to compare serum DHT levels in female and male patients with severe AS and to study its possible correlation to *1*) imaging parameters of LVH, fibrosis, and LV function before and after surgical aortic valve replacement (SAVR) and *2*) hypertrophy- and fibrosis-associated myocardial protein expression levels from LV biopsies.

## METHODS

### Patient Cohort

Sixty patients with severe AS and an indication for SAVR, according to current diagnostic guidelines (13), were prospectively enrolled within the SMART study (Systems Medicine of Heart Failure, clinicaltrials.gov NCT03172338). Exclusion criteria were the presence of moderate to severe aortic valve regurgitation (AR) and the presence of coronary artery disease with an indication for revascularization and general contraindications to cardiac magnetic resonance imaging examination (MRI). Within this cohort, 43 patients [median age 68 (41–80) yr] had received serum DHT level measurement from peripheral venous blood samples before SAVR. These patients were included in the present study. Patient characteristics are shown in Table 1.

**Table 1.** Patient characteristic

Preoperative Parameters	Aortic Valve Stenosis
*n*	43
Age, yr	66 ± 10
Body surface area, m^2^	1.98 ± 0.2
Females, *n* (%)	15 (35)
Systolic blood pressure, mmHg	138 ± 16
Diastolic blood pressure, mmHg	75 ± 9
Hypertension, *n* (%)	20 (47)
Dyslipidemia, *n* (%)	9 (21)
Diabetes mellitus, *n* (%)	3 (7)
Coronary artery disease, *n* (%)	0 (0)
Atrial fibrillation paroxysmal, *n* (%)	1 (2)
Atrial fibrillation permanent, *n* (%)	0 (0)
Left ventricular end-diastolic volume, mL/m^2^	83 ± 21
Left ventricular myocardial mass, g/m^2^	76 ± 25
Mean pressure gradient aortic valve, mmHg	53 ± 13
Aortic valve insufficiency, grade (none/mild, moderate, severe)	(43, 0, 0)
Left ventricular ejection fraction, %	56 ± 6
Fibrous tissue content, mL/m^2^	16 ± 5
Serum dihydrotestosterone level, pg/mL	244 ± 137
Medications	
ACE inhibitor, *n* (%)	5 (12)
β-Blocker, *n* (%)	6 (14)

Values are means ± SD or *n* (%); *n*, number of patients. ACE inhibitor, angiotensin-converting enzyme inhibitor. Mean pressure gradient aortic valve describes severity of aortic valve stenosis.

Routine clinical assessment in these patients included Doppler echocardiography with measurement of mean gradient across the diseased aortic valve (5-chamber view). Next to routine clinical examinations before SAVR, all patients underwent blood evaluation including DHT and estradiol levels and cardiac MRI. Blood pressure measurements were performed at the end of the MRI examination lying horizontally in supine position. The medical history of all patients was recorded, and cardiac MRI examinations were performed median 1 day before and median 110 days after SAVR. During SAVR of the 43 patients, LV myocardial samples were collected from *n* = 28 patients and used for proteomic profiling. In the remaining 15 patients, myocardial samples during SAVR could not be obtained because of technical reasons. Patient characteristics of the 28 patients were similar to the total cohort [mean age, 68 yr; body surface area (BSA), 2.0, female, *n* = 9 (39%); systolic blood pressure, 139 mmHg; diastolic blood pressure, 74 mmHg; mean pressure gradient across the aortic valve, 53 mmHg; and serum DHT level, 230 pg/mL].

The study protocol was in agreement with the principles outlined in the Declaration of Helsinki and was approved by the Medical Ethics Review Committee. All patients gave written, informed consent before inclusion.

### Cardiac Magnetic Resonance Imaging

#### Left ventricular mass, volume, and function.

All MRI examinations were performed using a whole body 1.5 Tesla MR system (Achieva R 3.2.2.0, Philips Medical Systems, Best, The Netherlands) using a five-element cardiac phased-array coil. Analyses were performed using View Forum (Philips Medical Systems Nederland B.V; View Forum R6.3V1L7 SP1). Gapless balanced Turbo Field Echo (bTFE) cine two-dimensional short-axis sequences were obtained using standard MRI protocol for LVM, volume, and function.

#### LV fibrous tissue content.

For fibrosis assessment, a single breathhold-modified Look-Locker inversion-recovery sequence in midventricular short-axis view was acquired before and 10 min after contrast administration. Calculation of extracellular volume (ECV) was performed using the following method:

$$ECV=\left(1-hematocrit\right)\;\text{×}\;\frac{(1/T\;myo\;post)-(1/T\;myo\;pre)}{(1/T\;blood\;post)-(1/T\;blood\;pre)}$$

where myo is LV midwall myocardial T1 value, blood is LV blood pool T1 value, and pre and post refer to the measurement before and after, respectively, contrast administration. Myocardial fibrous tissue content (absolute ECV = aECV) was calculated using the following equation: aECV = LV myocardial volume × ECV. LV myocardial volume = LVM/1.05, where 1.05 is the myocardial density (in g/mL).

### Global Longitudinal Strain Feature Tracking

MRI-based feature-tracking analyses were performed using commercially available software provided by Medis (QStrain, v. 2.1.12.2, Medis Medical Imaging Systems, Leiden, The Netherlands). Feature tracking was performed in the end-diastole and end-systole cardiac phases at the endo- and epicardial borders. Global longitudinal strain (GLS) was assessed by averaging the peak systolic strain values of 17 segments extracted from three LAX images (2-, 3-, and 4-long-axis CV).

### Circulatory Efficiency

Circulatory efficiency (CircE) describes the ratio between total left ventricular work and the work required for maintaining cardiovascular circulation and is defined as the ratio between circulatory power (CP) and left ventricular myocardial power (LVMP).

$$\text{CircE }=\frac{\text{CP}}{\text{LVMP}}$$

CP is defined as the hydrodynamic power distally to the valve representing the power needed to maintain effective blood flow against systemic vascular resistance (afterload)

$$CP\;=MAP\;\text{×}\;CO_{eff}$$

where MAP is mean arterial pressure and CO_eff_ is effective cardiac output. The dimension of CO_eff_ is liters per minutes. CO_eff_ is the product of heart rate and SV_eff_. SV_eff_ = (EDV − ESV) × (1 − regurgitation fraction), where SV_eff_ is effective stroke volume.

LVMP was defined as the surrogate power of the LV to perform one heartbeat since the applied method is an estimation.

$$LVMP\;=\frac{V_{wall}\;\text{×}\;\sigma_{wall}}{t_{CS}}$$

V_wall_ is myocardial wall volume, σ_wall_ is wall stress, and *t*_CS_ is LV systolic contraction time.

Wall stress was calculated using a simplified approach of the law of Laplace:

$$\sigma_{wall}\;=P_{sys}\;\text{×}\;\frac{R_{BP}}{2\;\text{×}\;S_{wall}}$$

where P_sys_ is LV peak systolic pressure, *R*_BP_ is mean radius of the blood pool, and *S*_wall_ is mean myocardial wall thickness. *S*_wall_ and *R*_BP_ during systole were averaged from LV segmentations considering the LV as a cylindrical geometry for correction of potential regional differences. P_sys_ is the sum of the systolic blood pressure measured at the right arm and the maximum pressure gradient across the aortic valve. LVMP was indexed to body surface area (BSA). All parameters were computed from cardiac magnetic resonance imaging-derived volumetric data and echocardiographic and clinical data. More details are described in former studies (14).

### Laboratory Testing

Peripheral venous blood samples were collected at time of MRI (around 5:00 pm in the afternoon) median 1 day before SAVR. DHT and estradiol levels were measured from serum blood tubes. Immediately after blood collection, blood samples were centrifuged and stored at 2°C–8°C for a maximum of 3 days. The concentration of DHT was determined by radiological immunoassay, and concentration of estradiol was quantified by electrochemical luminescence immunoassay as standardized measurements performed in the hospital-associated laboratory.

### Proteome Measurement

#### Sample preparation for mass spectrometry measurements.

*LV myocardial samples.* LV myocardial samples were collected from 28 patients with AS during aortic valve replacement surgery. Samples were taken and frozen directly in liquid nitrogen and kept at −80°C. Detailed information concerning protein extraction and peptide preparation, as well as the generation of a heart reference sample for matching library can be found in a recent publication (15). Protein concentration was measured using Bio-Rad DC Protein assay, and 100 µg of each sample was further processed using the SP3 cleanup and digestion protocol as previously described (16).

*LC-MS/MS analyses.* Peptide samples were eluted from stage tips (80% acetonitrile, 0.1% formic acid), and after evaporating, organic solvent peptides were resolved in sample buffer (3% acetonitrile/0.1% formic acid). Peptide separation was performed on a 20-cm reversed-phase column (75-µm inner diameter, packed with ReproSil-Pur C18-AQ; 1.9 µm, Dr. Maisch GmbH) using a 200-min gradient with a 250 nL/min flow rate of increasing *buffer B* concentration (from 2% to 60%) on a high-performance liquid chromatography (HPLC) system (ThermoScientific). Peptides were measured on an Orbitrap Fusion (individual samples) and Q Exactive HF-X Orbitrap instrument (reference sample) (ThermoScientific). Detailed information can be found in a recent publication (15).

MaxQuant software package (v1.6.2.6) was used to analyze the data. The internal Andromeda search engine was used to search MS^2^ spectra against a decoy human UniProt database (HUMAN.2019-01, with isoform annotations) containing forward and reverse sequences.

A deep-heart proteome dataset was generated from a mixed reference sample. Using two-dimensional liquid chromatography before tandem mass spectrometry analysis, we identified, in total 8,365 distinct protein groups. This deep reference proteome was used, as described by Doll et al (17), to match MS2 identification across individual runs to peptide precursors and to reach a uniform coverage across all samples with an average of 3,561 ± 187 proteins quantified per individual sample.

MaxQuant results were filtered to exclude reverse database hits, potential contaminants, and proteins only identified by site. All proteins for which the lead entry was marked “fragment” or with less than 50% valid values were excluded. Label free quantification (LFQ) values were log_2_ transformed, and missing values were imputed by random draw from Gaussian distribution with a downshift of 1.8 × standard deviation and 0.3 × standard deviation of the observed values per sample.

Analyses with regard to associations between myocardial protein expression levels and serum DHT levels and cardiac imaging parameters were performed for targeted proteins known to play a role in cardiac hypertrophy and fibrosis and partly known to be influenced by DHT.

### Statistical Analysis

In the first step, we performed a univariate correlation analysis of the clinical parameters using pairwise complete observations. All parameters that were found significant in univariate correlation analyses and which we considered clinically relevant and/or causally related to LVH were included in the final multiple linear regression model to predict indexed myocardial mass.

Proteins known to play a role in cardiac hypertrophy and fibrosis or known to be influenced by DHT were selected from the proteomic dataset. Associations between myocardial protein expression levels and serum DHT levels and cardiac imaging parameters were calculated in pairwise univariate analyses.

In all correlation analyses, we report the Pearson correlation coefficient as *R* and consider a *P* value of <0.05 as a significant finding. All statistical analyses were performed using R version 4.0.4.

## RESULTS

### Serum DHT Levels Correlate with Adverse Structural and Functional Myocardial Remodeling in Patients with Severe AS

**Figure 1.**
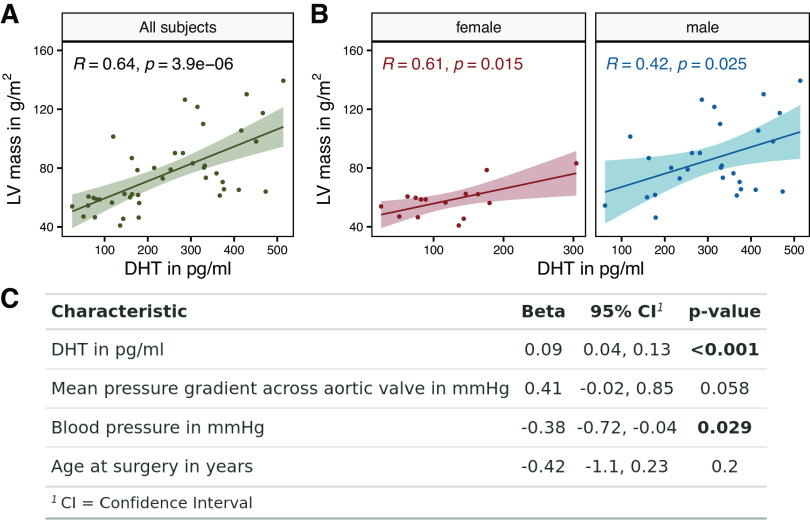
Serum dihydrotestosterone (DHT) levels correlate with left ventricular (LV) muscle mass. *A* and *B*: scatter diagrams with regression lines and 95% confidence intervals representing the correlation between serum dihydrotestosterone (DHT) levels and left ventricular (LV) muscle mass in all subjects and shown separately for female and male patients. *C*: multiple linear regression was calculated to predict indexed myocardial mass based on serum DHT levels, the mean pressure gradient across the stenotic aortic valve, the systolic blood pressure, and age at surgery. All subjects: *n* = 43, female: *n* = 15, male: *n* = 28, *R*, Pearson correlation coefficient.

Serum DHT levels correlated positively with LVMi (*R* = 0.64, *P* = 0.0001) in patients with severe AS, which was also present when looking at the subgroups of female and male patients separately (female, *R* = 0.61, *P* = 0.015; and male, *R* = 0.42, *P* = 0.025) (Fig. 1, *A* and *B*). Multiple linear regression was calculated to predict LVMi based on serum DHT levels, the mean pressure gradient across the stenotic aortic valve, the systolic blood pressure, and age at surgery. A significant regression equation was found [*F*(4, 35) = 10.09, *P* < 0.00005], with an *R*^2^ of 0.54. Coefficients, confidence intervals, and *P* values are listed in Fig. 1*C*. Serum DHT level is a significant predictor of LVMi. There was no correlation between LVMi and serum levels of estradiol in patients with AS.

**Figure 2.**
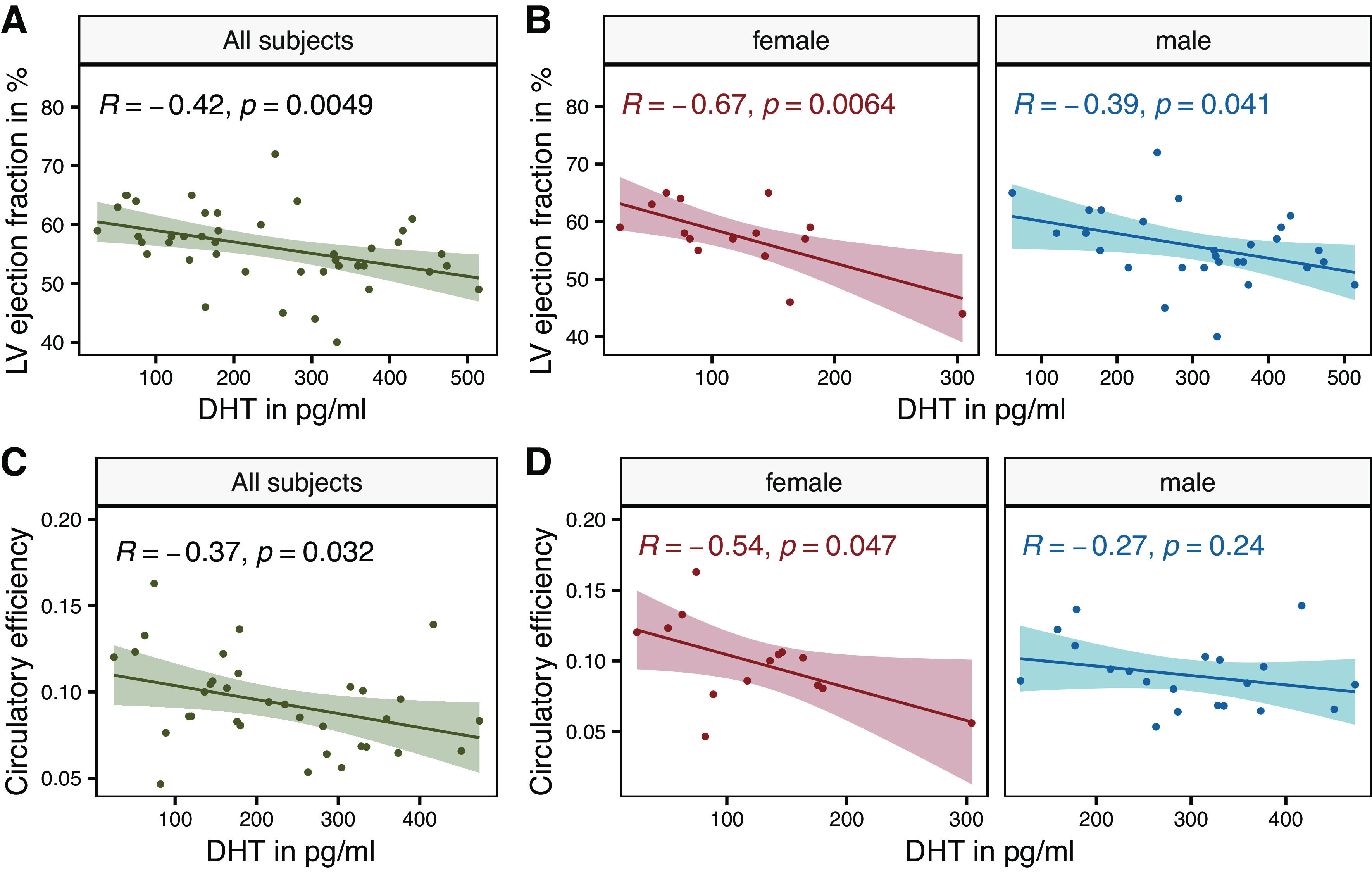
Serum dihydrotestosterone (DHT) levels correlate with left ventricular (LV) function. *A* and *B*: scatter diagrams with regression lines and 95% confidence intervals representing the correlation between serum dihydrotestosterone (DHT) level and left ventricular (LV) ejection fraction in all subjects and in female and male patients separately. *C* and *D*: circulatory efficiency correlates with serum DHT levels in all subjects and in female patients separately. All subjects: *n* = 43, female: *n* = 15, male: *n* = 28, *R*, Pearson correlation coefficient.

Serum DHT levels correlated inversely with LV ejection fraction (*R* = −0.42, *P* = 0.005) in the total cohort and within the subgroups of female and male patients separately (female, *R* = −0.67, *P* = 0.0064; and male, *R* = −0.39, *P* = 0.04) (Fig. 2, *A* and *B*). Serum DHT levels also correlated inversely with circulatory efficiency (*R* = −0.37, *P* = 0.032), which is an imaging-based surrogate marker describing the ratio between left ventricular work and the work required to maintain cardiovascular circulation in all patients with severe AS and within the subgroups in female (*R* = −0.54, *P* = 0.047) but not in male (*R* = −0.27, *P* = 0.24) patients (Fig. 2, *C* and *D*). Serum DHT levels additionally correlated with the clinically relevant biomarker nt-pro-BNP. There was no correlation between LV ejection fraction and serum levels of estradiol in patients with AS.

### Serum DHT Levels Correlate with Proteins Related to Cardiac Hypertrophy and Fibrosis

**Figure 3.**
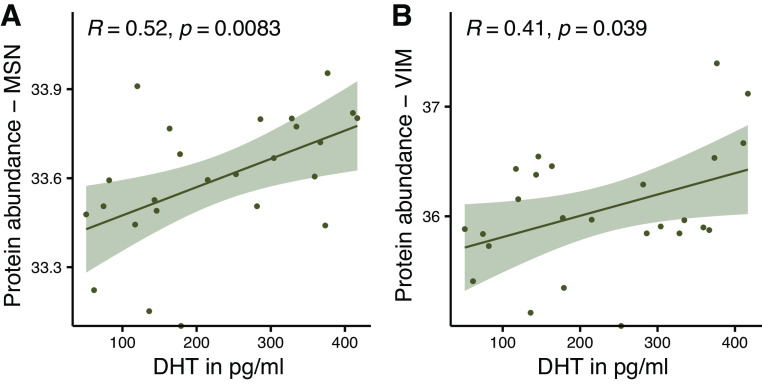
Serum dihydrotestosterone (DHT) levels correlate with prohypertrophic and profibrotic proteins. Scatter diagrams with regression lines and 95% confidence interval representing the correlation between serum dihydrotestosterone (DHT) levels and protein abundance of moesin (MSN; *A*) and vimentin (VIM; *B*) from left ventricular myocardial biopsies. MSN: *n* = 25, VIM: *n* = 21, *R*, Pearson correlation coefficient.

**Figure 4.**
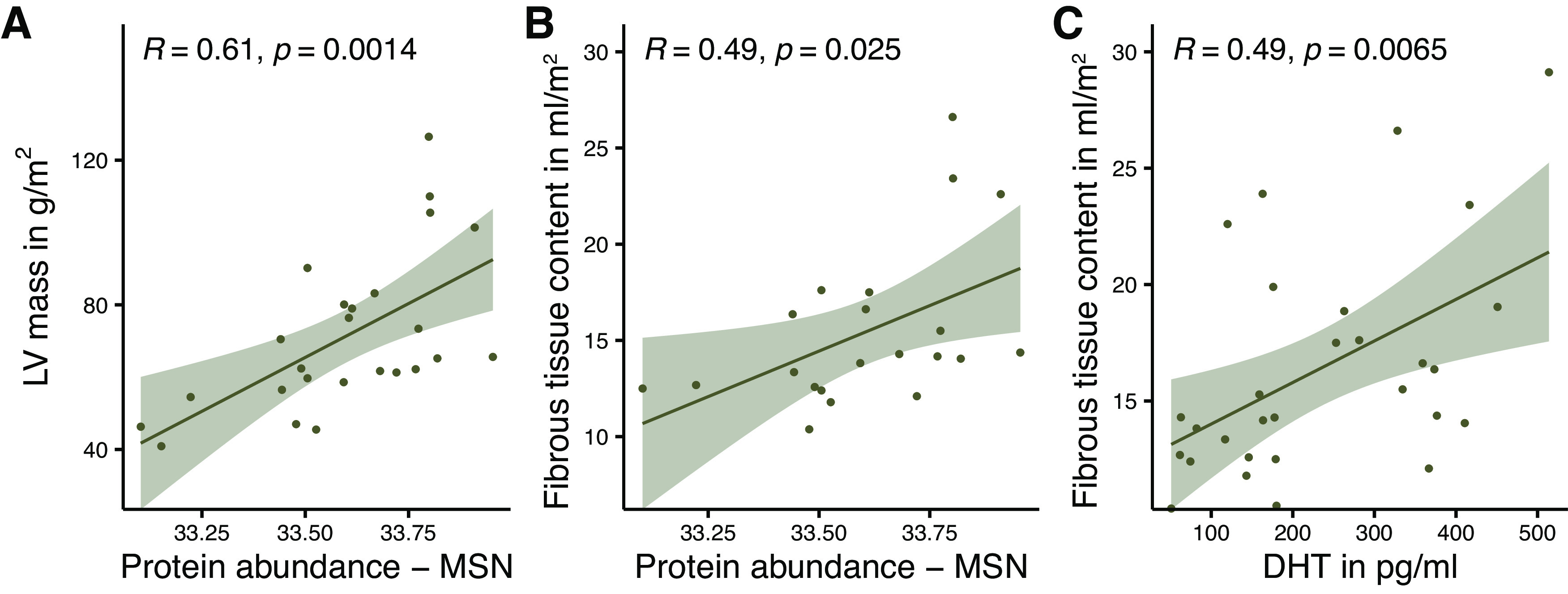
Correlation between serum dihydrotestosterone (DHT) levels and myocardial protein abundance with clinical parameters for hypertrophy and fibrosis from magnetic resonance imaging. Scatter diagrams with regression lines and 95% confidence intervals representing the correlation between protein abundance of moesin (MSN) and left ventricular (LV) muscle mass (*A*) and fibrous tissue content (*B*). Serum dihydrotestosterone (DHT) levels correlate with fibrous tissue content (*C*). *A*: *n* = 25, *B*: *n* = 21, *C*: *n* = 28, *R*, Pearson correlation coefficient.

**Figure 5.**
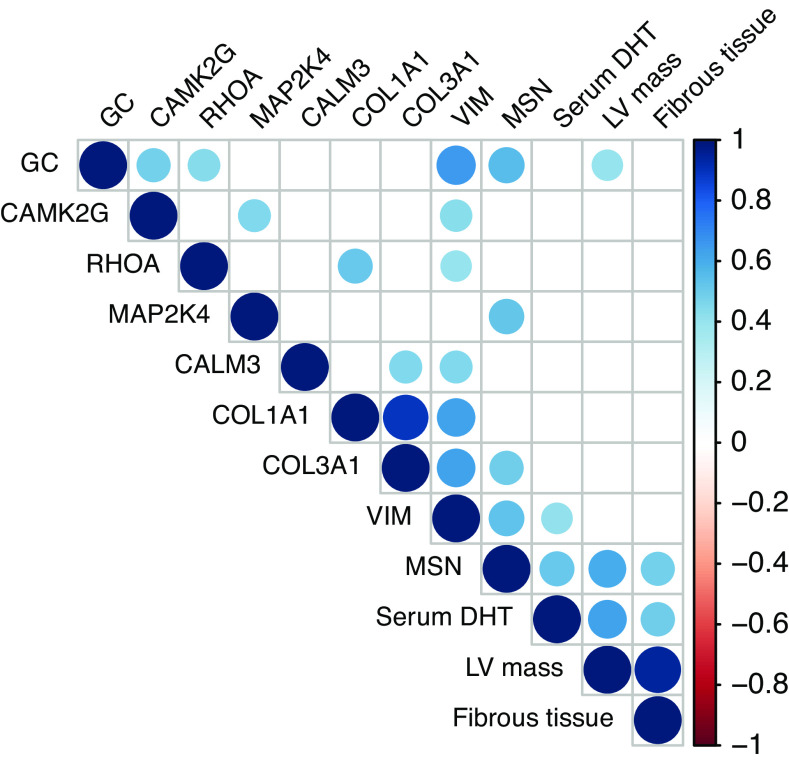
Correlation between serum dihydrotestosterone (DHT) levels, clinical imaging parameters, and protein abundances of selected hypertrophy- and fibrosis-associated proteins. Blue dots represent significant (*P* < 0.05), positive pairwise Pearson correlation coefficients. CALM3, calmodulin-3; CAMK2G, calcium/calmodulin-dependent protein kinase II-γ; COL1A1, collagen type I α1 chain; COL3A1, collagen type III α1 chain; GC, guanylate cyclase; MAP2K4, mitogen-activated protein kinase kinase 4; MSN, moesin; RHOA, Ras homolog family member A; VIM, vimentin.

Serum DHT levels correlate positively with the abundance of the prohypertrophic-acting protein moesin (MSN) (*R* = 0.52, *P* = 0.0083) and profibrotic-acting protein vimentin (*R* = 0.41, *P* = 0.039) (Fig. 3, *A* and *B*). Next to the already-described correlation between DHT and LVM, MSN also correlates with LVM (Fig. 4*A*) and, furthermore, both MSN and serum DHT correlate with clinical imaging parameter of fibrosis (*R* = 0.49, *P* = 0.025; and *R* = 0.49, *P* = 0.0065) (Fig. 4, *B* and *C*). Figure 5 shows all proteins that have been analyzed for a possible correlation with DHT.

### Higher Serum DHT Levels Preoperatively Are Associated with Reduced Myocardial Function and Increased Fibrosis after SAVR

**Figure 6.**
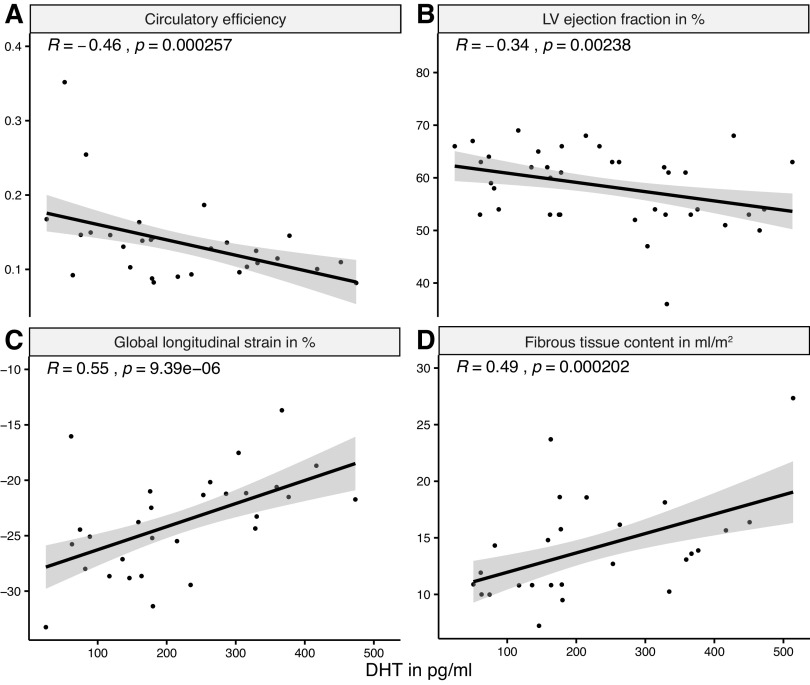
Preoperative serum dihydrotestosterone (DHT) levels in patients with aortic valve stenosis correlate with clinical parameters of left ventricular function and fibrosis after aortic valve replacement. Scatter diagrams are shown with regression lines and 95% confidence intervals representing the correlation between preoperative serum dihydrotestosterone (DHT) levels and clinical parameters of left ventricular function: circulatory efficiency (*A*), left ventricular ejection fraction (*B*), global longitudinal strain (*C*), and fibrosis (*D*) after aortic valve replacement. *A*: *n* = 27, *B*: *n* = 38, *C*: *n* = 29, *D*: *n* = 26, *R*, Pearson correlation coefficient.

Preoperative serum DHT levels correlated negatively with LVEF (*R* = −0.34, *P* = 0.035) and circulatory efficiency (*R* = −0.46, *P* = 0.012) and positively with global longitudinal strain (*R* = 0.49, *P* = 0.01) and fibrous tissue content (*R* = 0.55, *P* = 0.0022) after SAVR (Fig. 6).

### Antiandrogen Therapy is Associated with Low DHT and Favorable Cardiac Structure and Function in Patients with Severe AS

In our cohort, two male patients happened to have a medical history of prostate cancer and antiandrogen therapy (GnRH analog, central blockage of testosterone production) and presented with low levels of DHT (62 pg/mL, 179 pg/mL vs. median 329 pg/mL in all male patients), low LVMi (46 g/m^2^, 55 g/m^2^ vs. median 81 g/m^2^ in all male patients), low fibrous tissue content (13 mL/m^2^, 13 mL/m^2^ vs. median 17 mL/m^2^ in all male patients), and high LVEF (62%, 65% vs. median 55% in all male patients). Another two male patients had a medical history of benign prostate hyperplasia and finasteride treatment, which is a peripheral DHT conversion blocker. One patient showed low DHT levels (178 pg/mL), low LVMi (62 g/m^2^), low fibrous tissue content (14 mL/m^2^), and preserved LVEF (55%), whereas the other one showed comparable DHT values to the rest of the male cohort (335 pg/mL) and LVM, which was higher than in the three other patients, however, still in normal range (73 g/m^2^) (normal range for LVMi in male patients is >35 yr, 42–78 g/m^2^) (18). LVEF was mildly decreased (53%) and fibrous tissue content was mildly increased (15 mL/m^2^). The four male patients all had severe AS (mean gradient across the aortic valve: 40 mmHg, 48 mmHg, 49 mmHg, and 53 mmHg, respectively).

## DISCUSSION

With this explorative observational study, we provide new insights into the association between higher serum DHT levels and higher degree of cardiac hypertrophy, fibrosis, and dysfunction in human patients with pressure overload due to aortic valve stenosis before and after SAVR. Furthermore, we describe a correlation between serum DHT levels and expression levels of myocardial proteins associated with hypertrophy and fibrosis.

### DHT and the Degree of Remodeling in Pressure Overload

Testosterone and its active metabolite DHT have been shown to induce cardiac growth and dysfunction in cell culture and animal models (2, 19–21). The enzyme 5α-reductase is responsible for the conversion from testosterone to DHT, and the 5α-reductase blocker finasteride, which is used for example in male patients with prostate disease, leads to a reduction of DHT serum levels and has been shown to attenuate pressure-induced cardiac hypertrophy in mice (2). In humans, testosterone serum levels were associated with higher LVM in male patients with type I diabetes (5). However, these patients did not suffer from cardiac pressure overload and/or pathological cardiac hypertrophy. In the present study, we demonstrate for the first time that DHT is associated with adverse myocardial remodeling in patients with severe AS and in a small group of patients, antiandrogen therapy was associated with favorable cardiac structure and function. In addition, higher preoperative serum DHT levels in patients with AS were associated with reduced myocardial function and increased fibrosis after SAVR. We therefore speculate that DHT may contribute to adverse outcomes in patients with severe AS.

In a large retrospective analysis, it was described that finasteride treatment for prostate disease in male patients with heart failure was associated with reduced LV septal thickness (6). Prohypertrophic mechanisms of testosterone, however, are not only interesting in male but also in female patients. Women with polycystic ovary syndrome, for example, suffer from hyperandrogenism and are known to have an increased risk for LVH (22) and also female mice with induced polycystic ovarian syndrome progressively developed cardiac hypertrophy (23). These data from the literature suggest that testosterone may be associated with LVH also independent of pressure overload.

### DHT and Myocardial Protein Expression Levels

At the molecular level, it is known that androgen receptors are present in human and animal cardiac myocytes (3) and that myocytes respond to androgens with a hypertrophic response (21). Thus, the cardiac muscle phenotype can directly be regulated by androgenic steroids. The use of anabolic steroids (as synthetic derivatives of the male sex hormones testosterone), for example, was also shown to be associated with cardiac hypertrophy in both, human and animals (24) and low levels of DHT are described to be protective against cardiac hypertrophy (25).

Our study showed that serum DHT levels correlated not only with clinical parameters of hypertrophy and fibrosis but also increased expression of hypertrophy (moesin)- and fibrosis (vimentin)-associated proteins in LV samples from patients with severe AS. In addition, a positive correlation between moesin and increased LVM, as well as increased fibrous tissue content, could be detected.

Moesin is part of the ezrin/radixin/moesin (ERM) complex and can be activated by the cardiac sarcolemmal Na^+^/H^+^ exchanger (NHE), which is associated with the development of LV myocyte hypertrophy in animal studies (26–31). In human umbilical venous endothelial cells, it could be demonstrated that testosterone induces androgen receptor-mediated time- and dose-dependent increase of moesin expression (32, 33). In line with these findings, moesin activation was inhibited by the addition of the androgen receptor antagonist hydroxyflutamide, emphasizing the biological effectiveness of testosterone via androgen receptor-mediated pathways (32).

Vimentin on the other hand is a cytoskeletal protein present in fibroblasts and myofibroblasts and influencing extracellular matrix composition (34). In a hormone-sensitive human tumor cell line, it was also shown that DHT enhanced the expression of vimentin (35).

In accordance with these findings from cell culture and animal studies, we can describe the association between higher serum levels of DHT, increased moesin and vimentin expression levels, and higher degree of cardiac hypertrophy and fibrosis in patients with severe AS. As we cannot demonstrate a causal relationship between DHT and cardiac hypertrophy and/or fibrosis, we might, however, hypothesize a DHT-mediated increase in moesin and vimentin expression, which in turn might be associated with LVH and fibrosis. Future studies in animals with reduced, normal, and increased DHT levels might help to analyze the causal effects of DHT on LVH and fibrosis.

### DHT, but Not Estradiol, Was Associated with Cardiac Remodeling in Elderly Patients with Aortic Valve Stenosis

High levels of the female sex hormone estradiol have been shown to attenuate pressure overload-induced hypertrophy in both humans and animals (36, 37). In mice models with induced chronic pressure-overload, male mice show a higher proportion of pathological cardiac hypertrophy than female mice (38). In our cohort of elderly patients with AS (median age, 68 yr), however, an association between serum estradiol levels and cardiac hypertrophy could not be described. Estradiol levels in postmenopausal women are known to be low and not rarely below the detection limit of regular laboratory tests (39). The same was true in our cohort, where 11/15 (73%) female patients had no detectable estradiol levels. Besides, there were only measurements of estradiol levels and not of estrone, the dominant form of estrogen, which may be altered in our predominantly postmenopausal female patients with AS. If there could also be a shift in the balance between DHT and estradiol/estrone levels within the patients that might further influence the pathogenesis of cardiac remodeling and AS remains a matter of speculation and cannot be answered based on our data.

### Antiandrogenic Therapy – A Treatment Option to Reduce Hypertrophy in Patients with Aortic Stenosis?

Data from cell culture, animal, and human studies, including our own, show that male sex hormones influence cardiac remodeling (3–5, 19, 21). As the degree of cardiac hypertrophy, fibrosis, and dysfunction is known to have a negative impact on patients’ morbidity and mortality, it seems tempting to think about the use of antiandrogenic therapy to prevent cardiac remodeling in patients with AS to improve outcome in these patients (40, 41). In animal studies, a positive effect of antiandrogen therapy (finasteride) on cardiac hypertrophy, fibrosis, and function could be described (2). As finasteride has the ability to reduce DHT and to inhibit signal transduction in cardiac myocytes, it might be a therapeutic option to reduce LVH. In our study cohort, only a very small group of four male patients happened to be on antiandrogenic therapy because of prostate disease, and next to low levels of DHT, these patients also showed low degree of adverse cardiac remodeling and preserved cardiac function. However, we must emphasize that the small number of these patients receiving antiandrogenic therapy is a study limitation and prohibits to draw causative conclusions. It remains to be demonstrated if targeting DHT might improve outcomes in patients with AS.

### Conclusions

Higher DHT levels were associated with adverse myocardial structure, function, and proteomic remodeling in patients with severe AS. Future studies are needed to determine whether targeting DHT is effective in improving outcomes in patients with AS.

## GRANTS

This work was supported by German Federal Ministry of Education and Research (BMBF) Grant 031A427A), ERA-CVD (SICVALVES), and by European Commission H2020 Program Grant 689617 (Brussels, Belgium). This work was also supported by the Deutsche Forschungsgemeinschaft (DFG, German Research Foundation) Grant SFB-1470, project Z03 (to T.K.) and B05 (to P.M.). Marie Schafstedde is participant in the Charité Digital Clinician Scientist Program funded by the DFG, the Charité Universitätsmedizin-Berlin, and the Berlin Institute of Health at Charité (BIH).

## DISCLOSURES

No conflicts of interest, financial or otherwise, are declared by the authors.

## AUTHOR CONTRIBUTIONS

S.N. conceived and designed research; M.S., P.M., T.K., M.K., M.L.K., M.Z., and S.N. analyzed data; M.S., P.M., V.R-Z., M.K., and S.N. interpreted results of experiments; M.K. and S.N. prepared figures; M.S., M.K. and S.N. drafted manuscript; M.S., J.N., F.B., C.K., P.M., V.R-Z., T.K., M.K., and S.N. edited and revised manuscript; M.S., J.N., F.B., C.K., P.M., V.R-Z., T.K., M.K., and S.N. approved final version of manuscript.
